# Modeling and Experimental Investigation of U-R Relationship of AA6061-T6 Tubes Manufactured via Free Bending Forming Process

**DOI:** 10.3390/ma16237385

**Published:** 2023-11-27

**Authors:** Ali Abd El-Aty, Cheng Cheng, Yong Xu, Yong Hou, Jie Tao, Shenghan Hu, Bandar Alzahrani, Alamry Ali, Mohamed M. Z. Ahmed, Xunzhong Guo

**Affiliations:** 1 Department of Mechanical Engineering, College of Engineering at Al Kharj, Prince Sattam Bin Abdulaziz University, Al Kharj 11942, Saudi Arabia; ba.alzahrani@psau.edu.sa (B.A.); moh.ahmed@psau.edu.sa (M.M.Z.A.); 2 Mechanical Engineering Department, Faculty of Engineering-Helwan, Helwan University, Cairo 11795, Egypt; 3 College of Material Science and Technology, Nanjing University of Aeronautics and Astronautics, Nanjing 211100, China; c_cheng@nuaa.edu.cn (C.C.); taojie@nuaa.edu.cn (J.T.);; 4 Shi-Changxu Innovation Center for Advanced Materials, Institute of Metal Research, Chinese Academy of Sciences, Shenyang 110016, China; yxu@imr.ac.cn; 5 Department of Materials Science and Engineering & RIAM, Seoul National University, Seoul 08826, Republic of Korea; yonghou@snu.ac.kr

**Keywords:** 3D free bending forming, aluminum alloy tube, *U-R* relation curve, finite element simulation, forming experiment

## Abstract

Forming tubes with various bending radii without changing the bending dies is much easier for the 3D free bending forming (FBF) process. In the 3D-FBF process, different bending radii were realized by adapting the eccentricities of the bending dies. The accuracy of the *U-R* curve is crucial for the precision forming of complex bending components. In this study, the *U-R* relation curve of the Al alloy tube with a specific friction coefficient, fixed geometry size, clearance between tubes, and bending die was fitted first based on the forming results of AA6061-T6 tubes under different eccentricities. Second, the *U-R* relationship curve based on the experiment is used to propose the *U-R* relationship’s mathematical formula based on many hypotheses. Finally, the modified *U-R* mathematical formula was applied in the finite element (FE) simulation and the actual FBF experiments for the AA6061-T6 Al alloy complex shape space bending members. The *U-R* relationship curve’s reliability was verified by comparing the simulation and experimental results. The results obtained from the modified *U-R* relationship align well with the FE modeling results and can be directly applied to the bending process for the intended components.

## 1. Introduction

As aerospace, nuclear energy, and precision engineering fields progress toward miniaturization, integration, and lightweight solutions, the use of thin-walled metal tubular structures, prized for their hollow configuration, has surged. These tubes play pivotal roles in transferring mediums such as fuel oil and cooling water across various applications, from aircraft hydraulic mechanisms and mini satellite propulsion systems to steam condensation channels in small modular reactors (SMRs) and cooling pipes in compact electronics [1,2,3,4,5].

To accommodate the complex and changeable arrangement of piping systems, thin-walled metal tubular members with small diameters and thin walls often require multiple bends in space, continuous curvature variations, and small bending radii. The key benefit of these hollow metal tubes is their contribution to reducing the overall weight of components [6,7,8,9]. These applications often require tubes with complex spatial shapes. Traditional bending forming methods, however, are typically limited to creating simpler axial shapes [9,10,11,12]. For instance, Ghiotti et al. [13] utilized rotary draw bending to manufacture high-strength metallic alloy tubes with a nominal mean curvature radius of 100 mm and a bending angle of 90°. Simonetto et al. [14] introduced a three-roll bending technique for in-plane variable curvature components. Song et al. [15] proposed a push-bending forming technology with granular media filler for bending thin-walled tubes with a minor relative bending radius. As product customization increases, the shape of bent tube components becomes more complex, making it challenging to achieve bending forming with traditional methods. However, a significant technological advancement in plastic forming called free-bending forming has emerged, enabling one-time clamping to manufacture bent tubes with complex spatial shapes [16]. Moreover, M. Murata et al. [17] concluded that bending and forming components with different curved shapes could be achieved by controlling the spatial motion trajectory of the die.

In the flexible bending of tubes, material properties and process parameters play a crucial role in determining the axis shape, dimensional accuracy, and overall forming quality, given the minimal constraints imposed by forming dies [18]. For example, Guo et al. [18] examined the interplay between the deflection of the bending die (U) and the tube’s bending radius (R). Their findings indicated that higher values of elastic modulus (E), density (ρ), and strain-hardening exponent (n) lead to smaller bending radii. Groth et al. [19] employed a knowledge-based engineering approach to predict and correct shape deviations in the bending process. They developed manufacture-oriented models to establish process parameters for achieving specific bending geometries. Guo et al.’s research [20] revealed that while the feeding speed has a negligible effect on tube thickness and cross-sectional deformation, a minor increase in the gap between the forming die and the tube can significantly alter the bending radius. Beulich et al. [21] focused on the precision of FE simulations in flexible bending, considering factors such as material models, mandrel structures, and friction conditions. They used a mandrel connected by a steel rope to reduce axial imbalance defects in thin-walled tubes. Zhang et al. [22] created a geometric database to characterize the shape features of transition sections in bent tubes using free-bending technology. This database aids in optimizing clamp motion, thus minimizing axis shape errors and enhancing the quality of the bent tubes. Vatter et al. [23] explored the effects of various technological parameters, including mechanism motion, friction coefficient, and machine tool stiffness, on the geometry of spiral bending components in the roll-push-bending process.

One of the most crucial flexible bending processes used in recent years to manufacture thin-walled convoluted tubes is FBF technology. The FBF process offers a distinct advantage by enabling the integral formation of a spatial axis with continuous variable curvature in a single operation. This approach effectively circumvents the challenges associated with complex tubular member formation using traditional bending and welding processes, which often entail cumbersome procedures and high mold costs [24,25]. The FBF process could realize different bending radii without changing the bending die. Furthermore, springback compensation is easily realized by using the calibrated trajectory parameters of the bending die. In addition, various complicated bent tubes are manufactured successfully using the FBF process [26]. Due to the technology’s many remarkable advantages, it has been widely used in aerospace, nuclear power, automobiles, ships, and many other engineering fields [27]. On the other hand, compared to rotary draw bending, which relies on mold constraints [28], the FBF process offers less constraint on the tube during bending. This leads to increased complexity in affecting factors. Additionally, forming tubes with small relative bending radii becomes particularly difficult. These challenges pose a major obstacle to the widespread adoption and utilization of the FBF process [29].

Currently, the FBF forming process equipment mainly includes three-axis, five-axis, six-axis, and even seven-axis components based on a parallel mechanism [27,28,29]. This technology encompasses a three-axis free-bending mechanism, which consists of five main parts: the bending die, spherical bearing, guiding, compacting, and propulsive mechanisms [27]. All FBF experiments focus on accurately controlling the bending die’s trajectory in the bending process [28]. The distance between the center of the bending die and the center axis of the propulsion section of the tube is called eccentricity *U* [29]. By actively controlling the size and direction of the eccentricity *U*, a bending moment with different sizes and directions can be applied to the tube so that the bending radius *R*, bending angle, and relative position relation between different bending segments of the components have great degrees of freedom [30]. Thus, the bending radius *R* decreases with increasing eccentricity *U*. Therefore, an accurate *U-R* relation is significant for realizing the FBF process, especially for complex bent components. The *U-R* relationship plays a pivotal role in ensuring the precision of the tube FBF process. When considering tubes with identical cross-sections, material properties emerge as the primary factor influencing the *U-R* relationship.

Until now, there have been limited studies on how material properties influence the *U-R* relationship in the FBF process of tubes. For instance, Gantner et al. proposed a theoretical framework between *U* and *R* based on free-bending kinematic and mathematical models [31]. Additionally, they pointed out that specific adjustments are necessary for each material type being bent, given the differences in springback behavior among various materials [32]. Murata et al. [33,34] obtained the *U-R* relationship of different materials through preliminary FBF experiments. However, the application of the *U-R* relationship curve was not shown in the actual FBF process of different materials. Li et al. [35] found that the bending curvature increases linearly with increasing eccentricity. Furthermore, the slope of the *U-1/R* curve increases with the decrease in distance between the bending die and the guider. They mentioned that any bending radius and angle discrepancies were likely attributed to springback after deformation. The existing studies have primarily focused on determining the *U-R* relationship for specific materials through experiments or simulations without delving into the impact of internal material factors on this relationship [36,37,38,39]. Li et al. [40] emphasized the critical role of the eccentricity of the bending die in determining the bending radius of tubes. Through bending tests conducted on various Q235 angle steel profiles and tubes, they achieved a minimum relative bending radius of 1.7D, surpassing earlier benchmarks [37]. Hashemi and Niknam [41] delved into the effects of several factors on the bending radius of rectangular tubes, including the eccentricity of the bending die, its structure, the clearance, and the bending speed.

**Figure 1:**
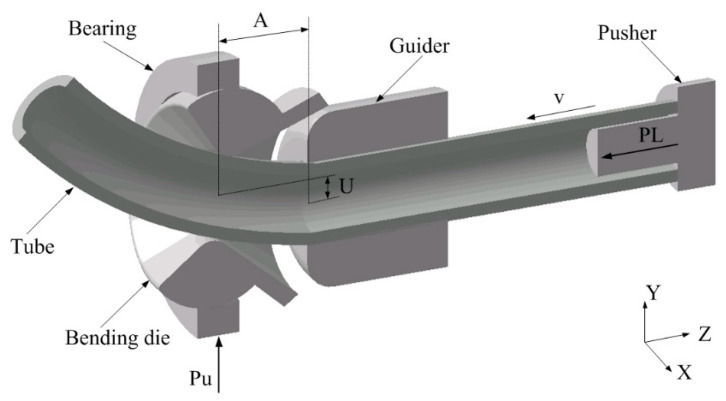
3D-FBF forming principle.

**Figure 2:**
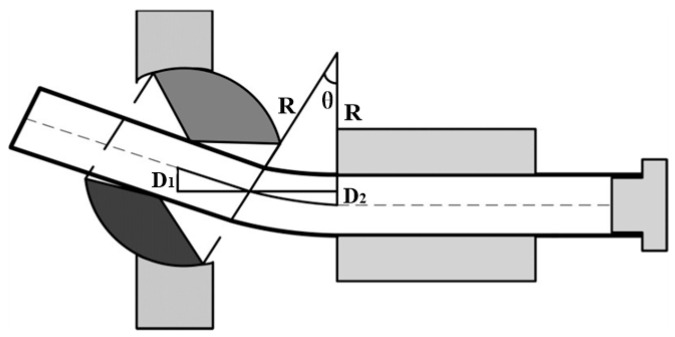
Transition section of the tube in the 3D-FBF process.

It is concluded from the discussion mentioned above that the material factors that affect the *U-R* relationship have not yet been investigated. Revealing how the material factors influence the *U-R* relationship is highly significant. It plays a crucial role in comprehending the forming mechanisms of the FBF process and is essential for predicting the *U-R* relationship for various materials. Thus, this study aims to propose a theoretical framework to determine the *U-R* relationship and consider the impact of material parameters on it. To propose a novel approach to determine the *U-R* relationship for the Al alloy (AA6061-T6) circular tube and examine the influence of material parameters on the *U-R* relationship, it is quite important to consider some assumptions mentioned in previous investigations [42,43,44,45]. These assumptions are generally adopted for the tube deformation process of the 3D FBF process, which is depicted in Figure 1. The first assumption is that the tube is deformed only in the forming zone and is kept straight in the guiding structure. Second, the reference axis of the bending die and the guiding mechanism is the theoretical central axis of the tube, without considering the dimensional tolerances between the bending die and the tube. Third, cross-section distortion did not occur in the forming process, and the tube’s curvature in the deformation zone was always constant. Finally, the springback of the bent tube after the unloading of the forming force was ignored. Based on the assumptions mentioned above, the transition section of the tube, which is depicted in Figure 2 in the forming process, is deduced. The tube in the deformation zone is composed of straight and arc sections. Nevertheless, the springback and the size change of the tube blank led to a specific deviation between the experimental results and theory prediction. To achieve good forming quality of tubes with different materials and sizes, the *U-R* relation curve must be obtained under specific tube forming test conditions. Thus, in this study, a novel approach is establishing the *U-R* relation for arbitrarily targeted Al alloy circular tubes. This method is developed through an analysis of how various material parameters influence the *U-R* relationship. The proposed *U-R* relationship closely aligns with those obtained from FE modeling and experimentation. In addition, it can be directly implemented in the bending program for the specific part intended to be bent. The process of predicting the *U-R* relationship was divided into three steps in the current investigation. Therefore, first, the *U-R* curve of the AA6061-T6 Al alloy tube under different bending radii was obtained through forming tests under different bending radii. Then, the *U-R* relation curve was used to modify the *U-R* mathematical model. Finally, the FE modeling and actual forming trials of the tube forming process were carried out using the modified *U-R* mathematical formula to verify the reliability of the *U-R* curve. The details of each stage will be discussed in the following sections.

## 2. *U-R* Relationship Obtained by the FBF Process

**Figure 3:**
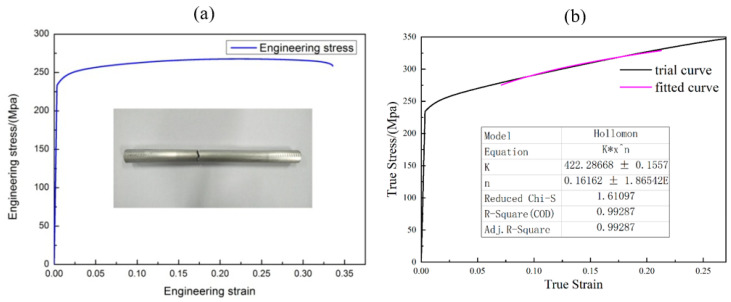
(**a**) Engineering and (**b**) true and fitted stress–strain curves of the AA6061-T6 Al alloy tube.

The outer diameter of the AA6061-T6 Al alloy tubes used in this study was 15 mm, and the wall thickness was 1 mm. The chemical composition of the AA6061-T6 Al alloy tubes is listed in Table 1. The mechanical properties of the tube were obtained by tensile testing using the MST-E45 universal testing machine. The tensile rate was set to 1 mm/s, the elongation was measured using the extensometer, and the engineering and trues stress–strain curves of the AA6061-T6 tube are depicted in Figure 3a,b, respectively. The mechanical properties of the rectangular AA6061-T6 tubes, which were obtained using curve fitting via the Hollomon constitutive model, are listed in Table 2.

**Figure 4:**
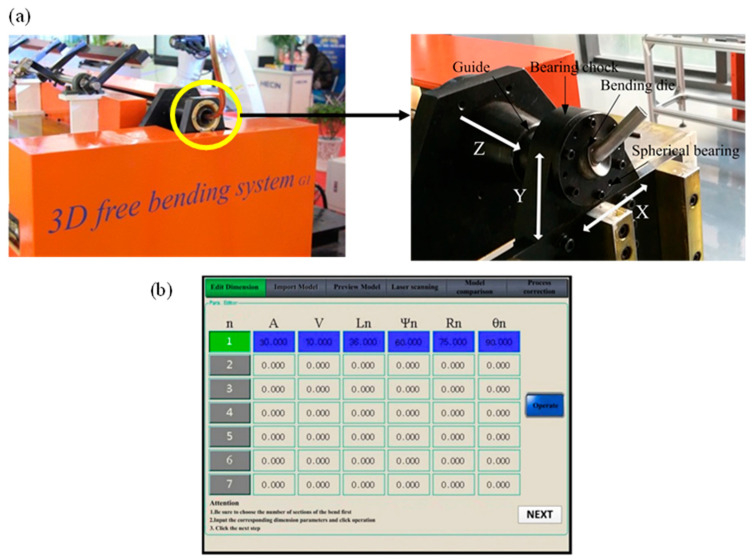
The developed three-dimensional FBF equipment. (**a**) The mechanical structure of the equipment. (**b**) The control software.

**Figure 5:**
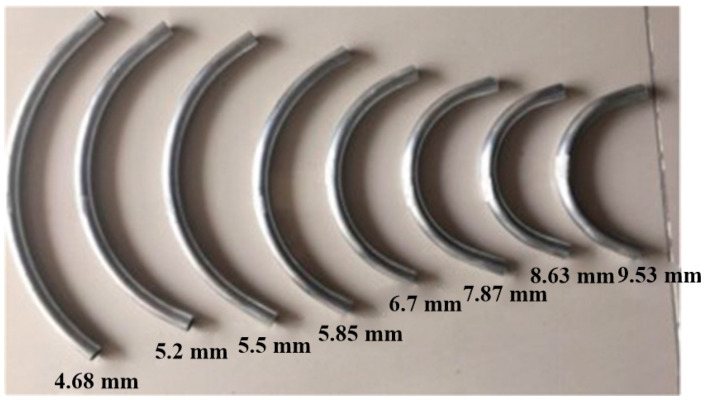
Forming results under different eccentricities.

**Figure 6:**
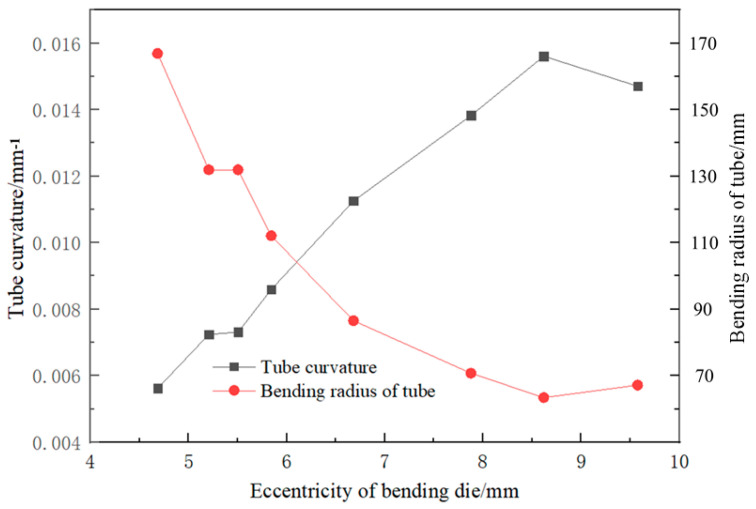
Relationship between bending radius R, bending curvature $\frac{1}{R}$ and eccentricity U.

The FBF test was carried out on self-developed three-dimensional FBF equipment, as shown in Figure 4a. Movement of the FBF equipment in the three directions of X/Y/Z was controlled in real-time by a X/Y/Z servo motor in three directions. During the forming process, the eccentricity of the bending die was set to 4.68 mm, 5.2 mm, 5.5 mm, 5.85 mm, 6.7 mm, 7.87 mm, 8.63 mm, and 9.57 mm. The friction condition between the tube and the forming part of the equipment was kept constant during the whole process. The distance between the die center and the leading end of the guider was 22.5 mm. The clearance between the tube and the die was 0.1 mm, and the feeding speed was 20 mm/s. The results of the bent tube corresponding to different eccentricities are shown in Figure 5. The relationship between the bending radius and the eccentricity of the tube is shown in Figure 6.

**Figure 7:**
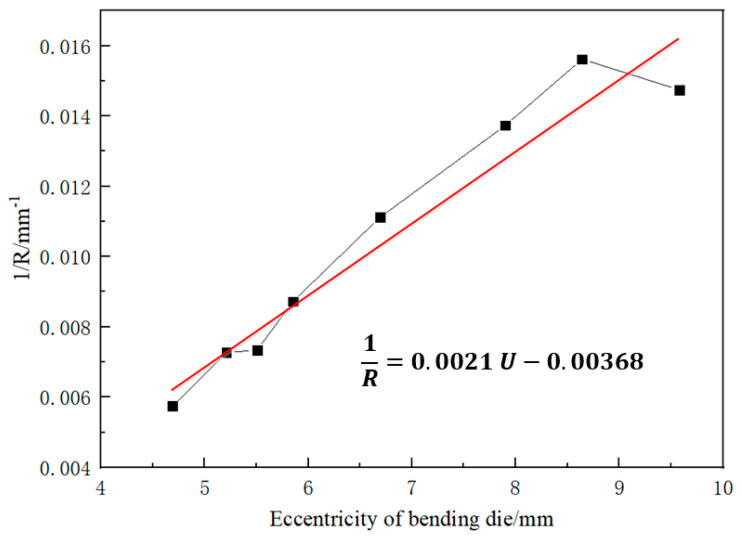
Fitting result of the $\frac{1}{R}-U$ relationship.

As seen in Figure 6, as the eccentricity of the bending die increases, the tube’s bending radius gradually decreases, and the reduced amplitude decreases gradually with increasing eccentricity. There is a near-linear relationship between $\frac{1}{R}$ and U. Therefore, the results obtained in Figure 6 were linearly fitted, and the result is shown in Figure 7. The correlation coefficient to judge the goodness of the fit is the fitting coefficient, which is determined by origin software.

## 3. Modification of the *U-R* Mathematical Formula

**Figure 8:**
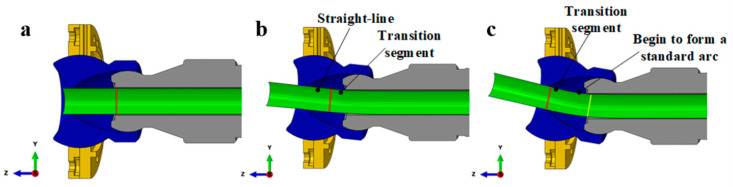
A schematic depiction of the bending die’s eccentric movement from its origin: (**a**) the die in its original position, (**b**) in the transition forming position, and (**c**) in the stable forming position.

In the tube-free bending system, the bending of any arc section involves three stages. First, the bending die transitions from a balanced position to an eccentric position, denoted as ‘upper *U*’, during which an arc length ‘upper *L*_1_’ is formed. The bending die remains stationary at the balanced position, resulting in an arc length ‘upper *L*_2_’. Second, the bending die returns from the eccentric position ‘upper *U*’ back to the balanced position, creating an arc length ‘upper *L*_3_’. Third, the design of the transition section is crucial for the accuracy of the bent tube. This transition section can be categorized into several key aspects. The study first focused on the length ‘upper *L*_1_’, examining the circumference at the outlet of the guider, as highlighted in red in Figure 8a–c. This analysis occurs under the combined influence of the upward movement of the bearing and the axial feeding direction. The tube section before this circumference remains a straight-line segment and is not considered a transition segment. In contrast, the tube section after the circumference forms a special-shaped arc segment. As the bearing moves upward, the circumference gradually nears the exit of the bending die. When it aligns with the die’s exit, a standard arc forms beyond the circumference. Once the bearing halts its upward movement, the bending of the standard arc segment commences. The transition segment ‘upper *L*_1_’ is located between the red and yellow circles in Figure 8c. This arc segment, being formed through a nonstandard process, is also referred to as the transition segment. Its length is determined to be a constant value.

Thus, *L*_1_ can be expressed as:

(1) $$L_{1}=\frac{\pi *R*\arcsin\left(A/R\right)}{180{}^{\circ}}$$

**Figure 9:**
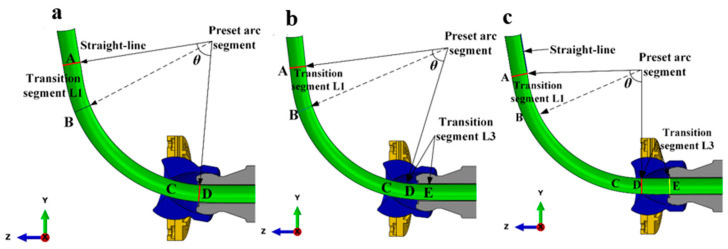
A schematic depiction of the bending die returning to the equilibrium position: (**a**) bending die positioned at its maximum eccentricity, (**b**) the bending die is in the process of returning to the equilibrium position, (**c**) the bending die is in the equilibrium position.

Second, the length of the transition segment *L*_3_, which is depicted in Figure 9, was examined. From Figure 9a, it is observed that the *AB* segment represents *L*_1_ of the transition section. After passing through *L*_1_, the tube begins to curve along a circular arc due to the axial feed force. This arc, with a preset angle *θ*, includes the circular arcs *AD* and *AD*, which are the target arcs for bending. At this stage, the bending die is positioned at *U*, its point of maximum eccentricity. The *CD* section remains within the bending die, where point *C* marks the die’s contact with the tube, and point *D* is where the tube exits the guider. Subsequently, as the bending die reverts to its balanced position from its maximum eccentricity, the tube continues its feed. This phase is depicted in Figure 9b, where the arc segment is *AD* and the tube progresses into the *DE* segment. Figure 9c illustrates that when point *D* aligns with the center of the bending die, the die simultaneously returns to its balanced position, marking the completion of the bending process for the arc with angle *θ*. The segment *DE* in this scenario constitutes the transition section *L*_3_. During the return of the bending die from its maximum eccentric position, its influence on the tube’s bending is minimal. Consequently, the *DE* section maintains a shape closely resembling a straight line. Upon the bending die’s return to the balanced position, the length of the transition section *L*_3_ is denoted as *A*.

**Figure 10:**
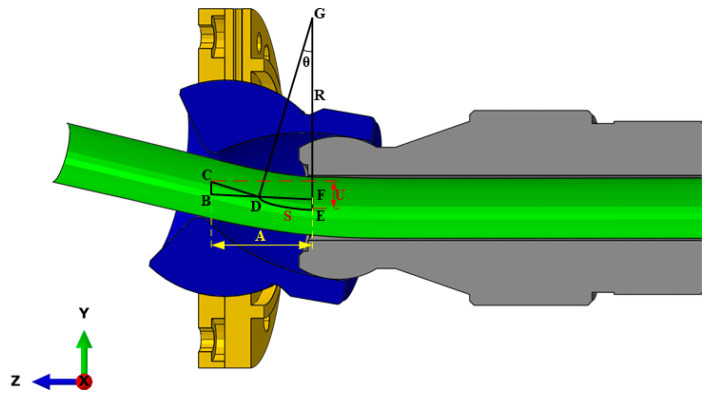
A schematic description of *L*_1_.

The feeding speed in the Z direction and the movement speed of the movable bearing on the X and Y planes need to be appropriately synchronized during different stages of bending. Consequently, the speed of the moving bearing is categorized into three stages: *L*_1_, *L*_2_, and *L*_3_. As depicted in Figure 10, when the bending die begins its movement toward the position with an eccentricity *U*, there is a combination of straight and arc segments ($BC+\hat{CE}$) between the bending die and the guider. Once the bending die arrives at the target position, the entire curve between the bending die and the guide comprises arc segments. This results in the transition segment and the transition segment reaching their maximum values. Thus, *U* is written as:

(2) $$U=BC+\hat{CE}=BC+\left(R-R\cdot \cos\theta \right)$$

In addition, Equation (2) can also be obtained from Figure 9 and written in another form as:

(3) A=BD+CF=BD+R·sinθ

By combining Equations (2) and (3) and the geometric relationship in Figure 9 and Figure 10, *U* can be expressed as:

(4) $$U=\left(A-R\cdot \sin\theta \right)\cdot \tan\theta +\left(R-R\cdot \cos\theta \right)$$

It can be seen from Figure 7 that the *U-R* relationship of the AA6061-T6 tube used in this test satisfies the relation $\frac{1}{R}=0.0021\;U-0.00368$. On the other hand, in the three-dimensional FBF system, the eccentricity *U* and the bending radius *R* satisfy the following equations under the condition of many assumptions:

(5) $$U=D_{1}+D_{2}=\left(A-R\cdot \sin\theta \right)\cdot \tan\theta +\left(R-R\cdot \cos\theta \right)$$

where $D_{1}$ represents the eccentricity of the straight section and $D_{2}$ represents the eccentricity of the arc section. Thus, Equation (5) can be used to describe the relation between U and *R*. For tubes of identical material and size, variations in *U* can be determined based on changes in *R* Equation (5). For ease of control, the Z direction is set for uniform motion with $\theta =\frac{180vt}{\pi R}$. Therefore, the following relationship can be obtained:

(6) $$U=\left(A-R\cdot \sin\frac{180vt}{\pi R}\right)\cdot \tan\frac{180vt}{\pi R}+\left(R-R\cdot \cos\frac{180vt}{\pi R}\right)$$

**Figure 11:**
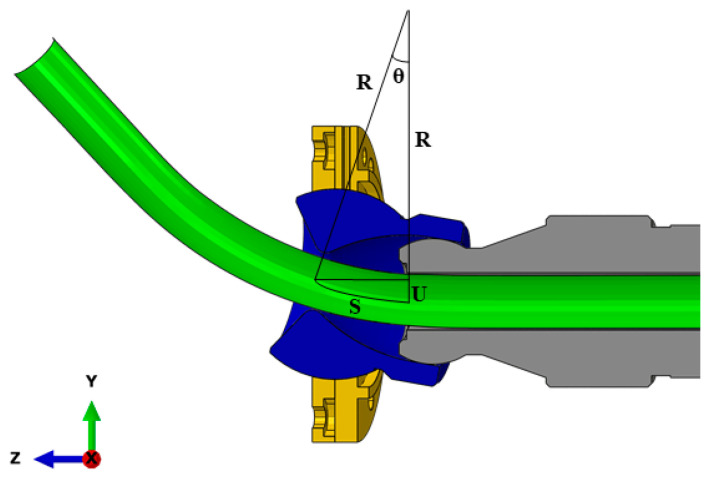
A schematic description of *L*_2_.

The $L_{2}$ phase is described in Figure 11, and $U=R-R\cdot \cos\frac{180S}{\pi R}$ is always constant; thus, the moving speed of the movable bearing in the $L_{2}$ stage is 0, and the eccentricity is unchanged.

**Figure 12:**
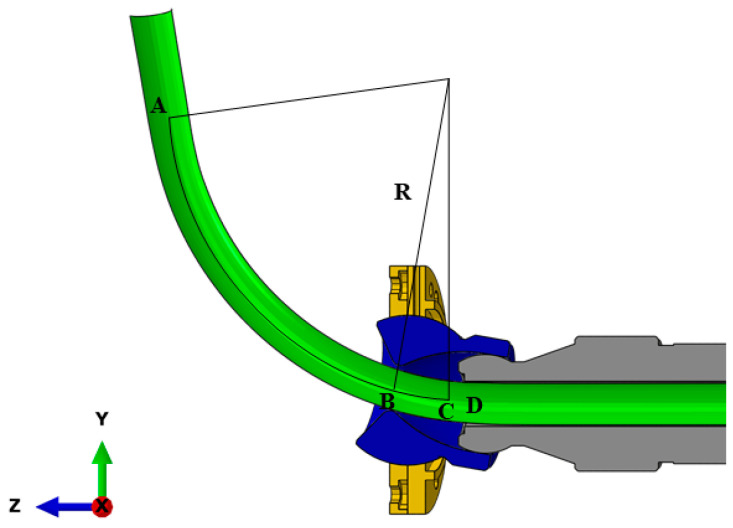
A schematic description of *L*_3_.

The *L_3_* phase is described in Figure 12. The AC segment is a preset arc segment with an angle of θ, the CD segment is a transition segment $L_{3}$, the *CD* segment is straight, and the *C* point is located on the *Z*-axis. Thus, the U corresponding to the $L_{3}$ segment is the projection length of the *BC* segment arc on the Y-axis. Thus, U can be written as:

(7) $$U=R-R\cdot \cos\theta =R-R\cdot \cos\frac{180BC}{\pi R}$$

The total arc length needed for the arc with a bending angle of θ is $\frac{\pi R\theta }{180}+A$, and the arc length of the *AD* section is variable *S*. Thus, the tube feed rate is $\left(\frac{\pi R\theta }{180}+A-S\right)$ to complete the arc bending process. Finally, point *C* reaches the center of the bending die, so the arc length of the *BC* section is $\left(\frac{\pi R\theta }{180}+A-S\right)$.

Thus, by combining all the abovementioned Equations and the geometric relationship in Figure 9, Figure 10, Figure 11 and Figure 12, $D_{1}$ and $D_{2}$ can be expressed as:

(8) $$D_{1}=\tan\frac{S\times 180^{{}^{\circ}}}{\pi \times R}\times \left(A-R\sin\theta \right)=\tan\frac{S\times 180^{{}^{\circ}}}{\pi \times R}\left(A-R\sin\frac{S\times 180^{{}^{\circ}}}{\pi \times R}\right)$$

(9) $$D_{2}=R-R\cos\theta$$

S represents the feed distance of the tube, described by v∗t. v is the axial feeding speed of the tube and t is the axial feeding time of tube $\left(0<t<\frac{\pi \times R\times \arcsin\left(\frac{A}{R}\right)}{180^{{}^{\circ}}\times v}\right)$. A is the distance between the center of the bending die and the front end of the guider. Thus:

(10) $$U=D_{1}+D_{2}=R-R\cos\frac{S\times 180^{{}^{\circ}}}{\pi \times R}+\tan\frac{S\times 180^{{}^{\circ}}}{\pi \times R}\left(A-R\sin\frac{S\times 180^{{}^{\circ}}}{\pi \times R}\right)$$

(11) $$=R-R\cos\frac{vt\times 180^{{}^{\circ}}}{\pi \times R}+\tan\frac{vt\times 180^{{}^{\circ}}}{\pi \times R}\left(A-R\sin\frac{vt\times 180^{{}^{\circ}}}{\pi \times R}\right)$$

In this experiment, v and A were considered to be 20 mm/s and 22 mm, respectively. Hence,

(12) $$U=R-R\cos\frac{20t\times 180^{{}^{\circ}}}{\pi \times R}+\tan\frac{20t\times 180^{{}^{\circ}}}{\pi \times R}\left(A-R\sin\frac{20t\times 180^{{}^{\circ}}}{\pi \times R}\right)$$

The axial feeding time of the tube (t) is defined by Equation (13) as:

(13) $$t=\frac{\pi \times R\times \arcsin\left(\frac{A}{R}\right)}{180^{{}^{\circ}}\times 20}$$

In this study, the distance A between the center of the bending die and the front end of the guider is multiplied by the correction coefficient k; thus,

(14) $$U=R-R\cos\frac{20t\times 180^{{}^{\circ}}}{\pi \times R}+\tan\frac{20t\times 180^{{}^{\circ}}}{\pi \times R}\left(kA-R\sin\frac{20t\times 180^{{}^{\circ}}}{\pi \times R}\right)$$

In addition, Equation (13) is expressed as:

(15) $$t=\frac{\pi \times R\times \arcsin\left(\frac{kA}{R}\right)}{180^{{}^{\circ}}\times 20}$$

For each specific bending radius $R_{0}$, the eccentricity $U_{0}$ obtained by equations 1/R=0.0021U−0.00368 and Equation (15) is equal to each other by adjusting the correction coefficient k. Then, Equation (15) is applied to the FBF simulation of the specific tube bending radius and actual forming process.

## 4. Verification of the Proposed *U-R* Relationship via FE Modeling and Experimentation

### 4.1. FE Modeling

**Figure 13:**
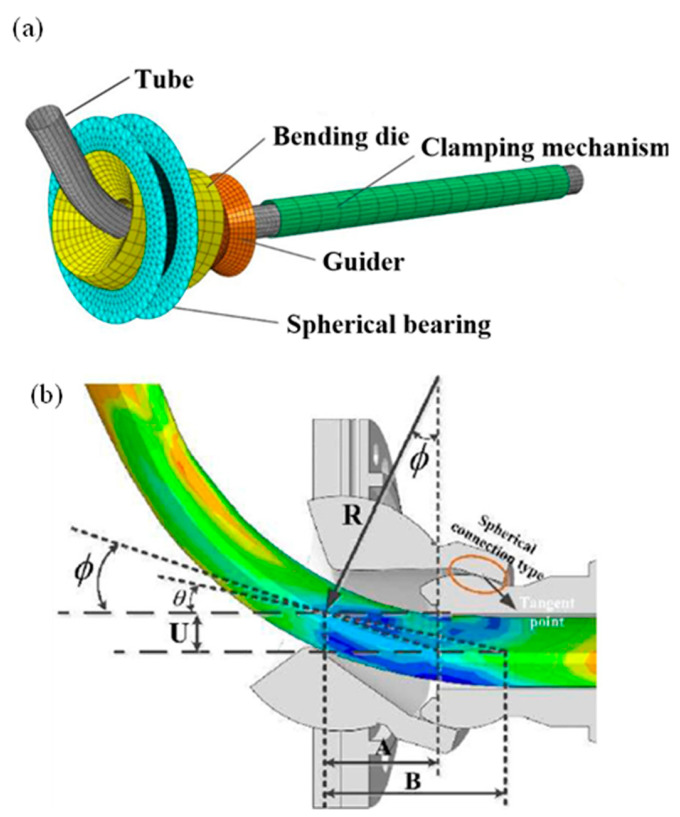
(**a**) FE model of the three-dimensional FBF process, (**b**) the spherical contact design of the guider and bending die.

The FE model of the 3D FBF process is shown in Figure 13. The model consists of five parts: the spherical bearing, bending die, guider, clamping mechanism, and tube. The tube and bending die were set to be deformable entities, and the grid type chose C3D8R, while other parts were set to be rigid bodies. The constitutive equation of the tube material is the Hollomon constitutive model described by $\sigma =K\varepsilon^{n}$, and the material parameters were set according to Table 2. The analysis step type was dynamically explicit. The interaction was set to be universal contact, and the friction coefficient took a value of 0.02. For load setting, the clamping mechanism and the guider were wholly fixed. The feeding speed in the Z direction was specified on the pusher. Speeds in the X and Y directions were applied on the spherical bearing.

**Figure 14:**
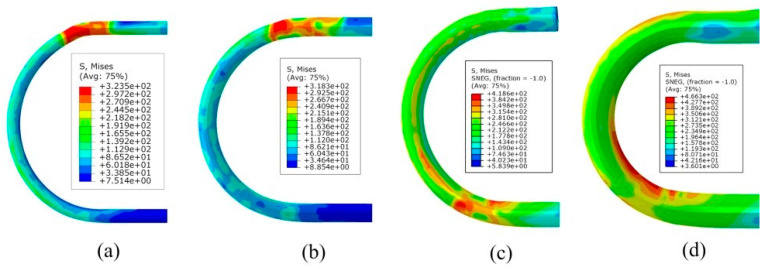
FE simulation results under different eccentricities. (**a**) *U* = 5.2 mm. (**b**) *U* = 6.7 mm. (**c**) *U* = 7.87 mm. (**d**) *U* = 9.57 mm.

**Figure 15:**
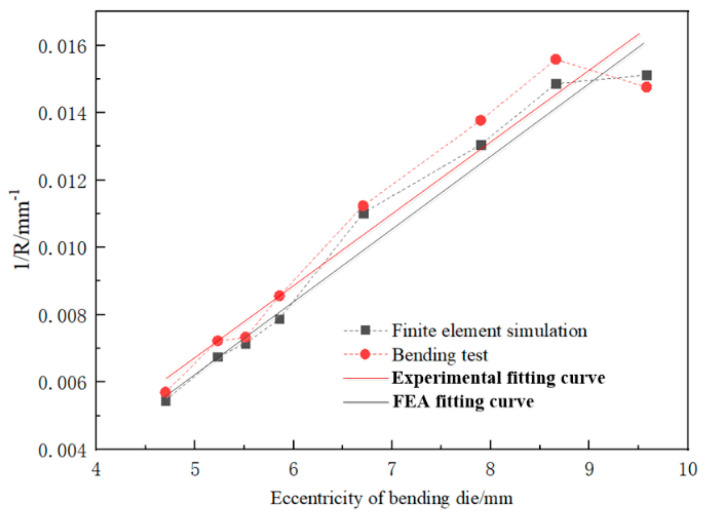
Comparison of experimental and simulated *U-R* curves.

To verify the accuracy of the FE modeling, this study used the built FE model to repeat the abovementioned bending tests. The FE modeling was carried out according to the eccentricity value used in the bending test. The results of FE modeling under different eccentricities are shown in Figure 14. The *U-R* relation curve obtained by FE modeling was fitted and compared with the *U-R* curve obtained by the actual trials, as shown in Figure 15. Within the allowable error range, the *U-R* relation curve obtained by FE modeling is very close to the experimental results, which verifies the reliability of the established FE modeling.

### 4.2. FBF Experiment of Complex Bend Components

**Figure 16:**
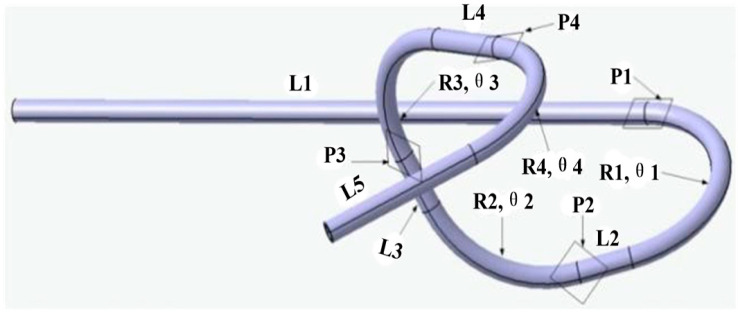
Three-dimensional geometrical model of the target tube.

To verify the reliability of the *U-R* curve, different eccentricity values corresponding to different bending radii were obtained by the *U-R* relation curve. In this study, the FE simulation and FBF experiment of the AA6061 alloy tube were conducted. Figure 16 shows the three-dimensional geometrical model of the target tube fitting, where *P1*~*P4* indicates four bending planes. The outer diameter of the target tube was 15 mm, and the wall thickness was 2 mm. The total axial length was 1558 mm. Specific dimensional parameters for each bending section are shown in Table 3, where $\psi_{n}$ is the direction of each bending section.

Based on the correction coefficient k obtained from Table 4, the matching relationship between the bending speed of the bending die and the feeding speed of the pusher was calculated. Then, the velocity-matching relationship and the eccentricity of the bending die were applied for the FE simulation and the FBF experiment. Table 5 shows the geometrical comparison result between the CAD model, FE simulation result, and forming result. It can be seen from Table 5 that the geometrical shapes are almost the same. Table 6 shows the geometric dimensional parameters, error comparison, maximum wall thickness reduction rate, and maximum cross-sectional distortion rate in each bending plane. It can be seen from Table 6 that the deviation of the tube bending radius is relatively small, and the maximum deviation does not exceed ±5%, which indicates that the obtained *U-R* relation curve of the AA6061-T6 Al alloy tube is accurate and reliable. However, the length of the straight section and the bending angle differ from the design dimension. This may be due to mechanical and measurement errors. The tube’s maximum wall thickness reduction rate is not more than 9%, and the maximum cross-section distortion rate is less than 5%, indicating a fine forming quality.

## 5. Conclusions

Based on the achieved results from the current study, the following conclusions can be deduced:

1. The modified *U-R* mathematical formula of the AA6061-T6 Al alloy tube is 1/R=0.0021U−0.00368 through the FBF experiment under different eccentricities and certain process conditions.
2. The FE simulation result of the *U-R* relation curve is close to the experimental result, proving that the result shows a good guide for the FBF experiment.
3. The FE simulation and the bending experiment were carried out based on the modified *U-R* relation curve. The results show that the experimental results are in good agreement with the CAD model and the simulation results. The test tube bending radius deviation is relatively small, and the maximum deviation is not more than ±5%, indicating that the *U-R* relationship of the AA6061-T6 Al alloy tube is accurate and reliable.
4. In the ideal forming process, the contact point between the bending die and the tube’s outer bend should remain tangential. However, due to factors such as the bending die clearance and material properties, the rotation angle can be adjusted within a certain range to still ensure smooth tube formation. Deviations from this tangential state, caused by changes in the rotation angle, can lead to overbending or underbending. Thus, it is quite important to propose a new theoretical analysis of the free bending process, taking into account the clearance to analyze the material flow and bending radius changes during nontangential contact between the bending die and the tube. This analysis is crucial to explaining why smooth tube bending is achievable despite rotation angle adjustments and examines the tube’s deformation mechanism under the combined influence of additional tangential force from the bending die and axial propulsive force.

## Figures and Tables

**Table 1:** The chemical composition of the AA6061-T6 Al alloy tube.

Ingredient	Si	Fe	Cu	Mn	Mg	Cr	Ti	Al
Content	0.72	0.52	0.3	0.15	1.1	0.3	0.05	Balanced

**Table 2:** The density and mechanical properties of the AA6061-T6 Al alloy tube.

Density *ρ* (g/cm^3^)	Poisson’s Ratio *ν*	Uniform Elongation Ratio *δ* (%)	Young’s Modulus *E* (GPa)	Yield Stress *σ_s_* (MPa)	Strength Coefficient *k*	Hardening Exponent *n*
2.75	0.33	16.59	69	237.39	422.29	0.1616

**Table 3:** Specific dimensions of the target tube.

Bending Plane	Straight Length *L_n_* (mm)	Bending Radius *R_n_* (mm)	Bending Angle *θ_n_* (°)	$\mathrm{Bending}\;\mathrm{Direction}\;\psi_{n}$ (°)
*P1*	600	77.5	137	—
*P2*	40	71	106	45
*P3*	40	68	157	45
*P4*	50/120	83	170	45

**Table 4:** Modification of the theoretical model.

Bending Radius *R*/mm	77.5	71	68	83
Eccentricity of bending die *U*/mm	7.89	8.46	8.76	7.49
Actual *A* value/mm	34.07	33.61	33.39	34.46
Theoretical formula correction factor *k*	1.514	1.494	1.484	1.532

**Table 5:** Geometrical comparison between the CAD model, FE simulation result, and forming result.

CAD Model	Finite Element Simulation Result	Forming Result
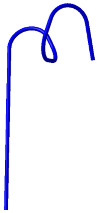	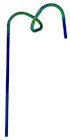	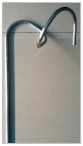
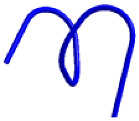	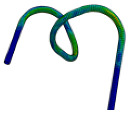	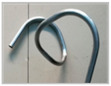

**Table 6:** Geometric parameters of the forming result.

Curved Plane	Straight Length *L_n_* (mm)	Bending Radius *R_n_* (mm)	Bending Angle *θ_n_* (°)	$\mathrm{Bending}\;\mathrm{Direction}\;\psi_{n}$ (°)	Maximum Wall Thickness Thinning Rate/(%)	Maximum Cross-section Distortion Rate/(%)
*P1*	—	81	120	—	6.57	4.45
Deviation/(%)	—	4.5	−12.4	—	—	—
*P2*	44	73	98	48	8.74	3.38
Deviation/(%)	10	2.8	−7.5	6.7	—	—
*P3*	42.5	70.5	145	43	7.65	4.89
Deviation/(%)	6.25	3.7	−7.6	−4.4	—	—
*P4*	53	80	156	46	8.21	4.14
Deviation/(%)	6	−3.6	−8.2	2.2	—	—

## Data Availability

Data will be available upon request through the corresponding author.
